# “Voice your choice”: a study of women’s choice of surgery for primary stress urinary incontinence

**DOI:** 10.1007/s00192-019-04202-6

**Published:** 2019-12-18

**Authors:** Lucy Dwyer, Emily Weaver, Azita Rajai, Samantha Cox, Fiona Reid

**Affiliations:** 1 The Warrell Unit, Saint Mary’s Hospital, Manchester University NHS Foundation Trust, Manchester Academic Health Science Centre, Hathersage Road, Manchester, M13 9WL UK; 2 Manchester University NHS Foundation Trust, Manchester Academic Health Science Centre, Hathersage Road, Manchester, M13 9WL UK; 3 Institute of Human Development, Faculty of Medical & Human Sciences, University of Manchester, Manchester Academic Health Science Centre, Oxford Road, Manchester, M13 9PL UK

**Keywords:** SUI, Choice, Decision making, Urethral bulking, Mid-urethral tape, Fascial sling, Colposuspension

## Abstract

**Introduction and hypothesis:**

This was an observational study aiming to determine factors which influence women’s choice of surgery for primary stress urinary incontinence (SUI).

**Methods:**

Two hundred twelve women undergoing a primary SUI procedure were recruited to this study from 12 hospitals in the north of England. After choosing a procedure, women were asked to complete a standardized semi-structured questionnaire about their health, demographics and a free text box to record factors important to them when choosing their procedure. Statistical analysis was performed to determine the impact of demographic, lifestyle or healthcare factors on women’s decision-making. Thematic analysis of the free text data was performed to identify factors important for women when choosing a surgical procedure.

**Results:**

Sixty-four percent of women chose urethral bulking. There was no significant difference among age, BMI, smoking status or previous laparotomy between women choosing the four types of surgery. Women were less likely to choose urethral bulking if seen in a tertiary centre compared with a secondary centre (*p* < 001). Major themes in decision-making were efficacy, invasiveness, recovery, risk of complications, use of mesh, the clinician, the media, hierarchy of treatments and type of anaesthetic. Some women expressed a hierarchical approach to treatment.

**Conclusions:**

Our findings suggest decision-making is not influenced by patient factors such as age, BMI, smoking status or previous laparotomies. Women’s choices are a complex mix of factors and not simply related to efficacy.

## Introduction

Stress urinary incontinence (SUI) is a very common condition. Procedures to treat SUI include urethral bulking, mid-urethral slings (MUS), autologous fascial slings and colposuspension. Over the last decade, continence surgery has been the subject of controversy in the UK, and there have been several government inquiries and reviews of safety, primarily in relation to mesh. The most recent inquiry in England, the Independent Medicines and Medical Devices Safety Review, was commissioned by the Government in February 2018 and has yet to report its conclusion. Until the 2018 pause on MUS in England, MUS were by far the most common procedure performed, with 84 tapes for every one non-tape procedure [1].

One of the recurring themes in these inquiries was the poor counselling provided by clinicians to women undergoing surgery for SUI. This has led clinicians to consider ways to improve the information they provide. The recently published NICE guideline on incontinence and prolapse [2] contains patient decision aids (PDA). However, it contains very little on how women can make a choice among three operations, with fairly similar outcomes. It also treats bulking as a separate category of treatment. Other PDAs for SUI have been published [3, 4]; however, these are based on expert opinion rather than a systematic meta-analysis of the evidence which NICE undertook. Furthermore, none of the PDAs were informed by how women chose an operation for SUI. It is important clinicians understand what matters to women considering treatment. Therefore, this study endeavoured to gain greater insight into the process of women‘s decision-making when choosing an SUI procedure. This knowledge may lead to patient-focused PDAs and may help to identify gaps in current evidence which require further research.

## Materials and methods

### Study design

Latitude is a multi-centre observational study to investigate the long-term effectiveness of the urethral bulking agent, Bulkamid®, as a primary treatment for SUI (ClinicalTrials.gov Identifier: NCT03474653). A descriptive questionnaire study was embedded within Latitude with the objective of exploring factors which influence patients’ choice of surgical treatment for SUI. Eligible participants were recruited between June 2017 and July 2018 from 12 sites in the north of England. It is important to note that data for this study were collected before NHS England paused the use of polypropylene mesh tapes for continence surgery [5].

All women who had made a choice about which SUI procedure they wanted were approached. The inclusion criteria required that women were having their first procedure for SUI, had stress predominant mixed urinary incontinence, no evidence of detrusor over-activity or voiding difficulties and were deemed suitable by the surgeon for at least two of four surgical options (urethral bulking, mid-urethral sling, colposuspension or fascial sling). The full eligibility criteria are detailed within Table 1. Participants were counselled in their routine clinical setting, there was no pre-determined script used by the clinician and options were presented according to routine clinical practice.

**Table 1:** Eligibility criteria

Inclusion criteria
• All women with urodynamic stress incontinence who are suitable for at least two SUI surgical procedures • Evidence of previous pelvic floor muscle training
Exclusion criteria
• OAB (overactive bladder) predominant mixed incontinence • Any previous surgery for urinary incontinence • Concomitant prolapse surgery • Detrusor over activity on urodynamics • Residual urine > 100ml at urodynamics • Bladder capacity < 300 ml • An acute urinary tract infection • An allergic reaction to the local anaesthesia used in the treating unit • An allergic reaction to all the antibiotics which could be used for prophylaxis • Current treatment with systemic corticosteroids • Pregnancy • Active autoimmune or connective tissue diseases • Not fluent in English and therefore unable to complete the study questionnaire • Lacking capacity to consent to participate in the study

Participants were asked to complete a semi-structured questionnaire (Appendix). The questionnaire recorded patient demographics, lifestyle and medical information, the surgical procedures discussed with the clinician and the surgical procedure the patient chose. In a free text box women were asked: “What was the most important issue to you when making your treatment decision?” In another free text box they were also asked if they would have liked any additional information to help them make that decision.

### Analysis

Analysis of the quantitative data collected was carried out using Rx64 3.5.1. Continuous variables were compared using the Mann-Whitney U test and categorical ones using the chi-squared test to explore whether factors such as age, BMI, smoking status, hospital type or history of laparotomies impacted upon patient decision-making.

To explore procedure choice, a free text response to an open question was chosen because the research team did not wish to presume what mattered most to women. Thematic analysis was used to identify, analyse and report themes within the free text data. Thematic analysis was conducted independently by two members of the research team blinded to the procedure chosen and all other patient data. Codes were then compared and rationalized and emerging themes identified [6]. The frequency of codes was also recorded.

## Results

Two hundred sixteen women completed the questionnaire. Four questionnaires were excluded from analysis because two women decided not to go ahead with their SUI treatment and another two were identified to be ineligible as this was not their first continence procedure.

The analysis of 212 women found 64% opted for urethral bulking (*n* = 135), 23% a mid-urethral sling (*n* = 48), 12% colposuspension (*n* = 25) and 2% a fascial sling (*n* = 4). Characteristics of the sample population are detailed within Table 2. There was no significant difference in age, BMI, smoking status or previous laparotomies and the procedure chosen for SUI (Table 3). Hospital type was a significant factor in determining the procedure chosen (*P* < 0.001) with mid-urethral sling and colposuspension or fascial sling all most likely to be chosen by women attending a tertiary hospital compared with those attending a secondary-care hospital (Table 3). However, at both hospital types urethral bulking was the most frequently chosen procedure (75% in secondary-care hospitals and 51% in tertiary hospitals).

**Table 2:** Characteristics of the sample population

	*n* = 212
Age	Media *n* = 50
	IQR = 44,58
	Range = 27–84
BMI	Media *n =* 28
	IQR = 25,32
	Range = 17–58
Smoking status	Yes *n* = 27 (13%)
	No *n* = 185 (87%)
Site type	Secondary *n* = 111 (52%)
	Tertiary *n* = 101 (48%)
Laparotomy	None *n* = 130 (62%)
	One *n* = 63 (30%)
	Two or more *n* = 19 (9%)
	Missing data *n* = 1

**Table 3:** Procedures chosen and participant demographics and lifestyle factors

*n = 212*		Mid-urethral sling *n* = 48 (23%)	Urethral bulking *n* = 135 (64%)	Fascial sling/ colposuspension *n =* 29 (14%)	*P* value
Age	Median	48	50	54	*P* < 0.73
	Range	31–81	27–84	30–71	
BMI	Median	27	28	27	*P* < 0.59
	Range	21–58	17–50	21–41	
Smoker	Yes	7 (15%)	18 (13%)	2 (7%)	*P* < 0.58
Hospital	Secondary	20 (42%)	83 (61%)	8 (28%)	*P* < 0.001
	Tertiary	28 (58%)	52 (39%)	21 (72%)	
Laparotomy	0	29 (60%)	84 (62%)	17 (59%)	*P* < 0.96
	1	15 (31%)	38 (28%)	10 (34%)	
	2 or more	4 (8%)	13 (9%)	2 (7%)	

Thematic analysis found the major themes associated with patient decision-making were as follows.

### Invasiveness of the procedure

Women’s concerns about the “invasiveness” of the procedure were expressed by 53 women (25%). While invasiveness was frequently cited as a factor which affected decision-making, only a small number of women defined what made them consider a procedure more or less invasive:

*‘Less cuts.’*

*(Chose urethral bulking)*

It was apparent from the analysis some women did not consider injection of a urethral bulking agent to be surgery:

*‘(I) feel that my condition isn’t severe enough for an operation.’*

*(Chose urethral bulking)*

*‘I was very relieved when…the bulking agent was offered, I have lived with symptoms for years thinking surgery was (the) only option.’*

*(Chose urethral bulking)*

Of the 53 women who reported invasiveness was an important factor in decision-making, 83% opted for urethral bulking (*n* = 44), 9% a colposuspension (*n* = 5) and 8% a mid-urethral sling (*n* = 4).

### Chance of success

Success rate and “invasiveness” of the procedure were jointly the most frequently mentioned factors in women’s decision-making. Fifty-three women, 25% of the total cohort, reported the success rates of different procedures influenced their choice. Reassuring statistics about likelihood of cure were of overriding importance as illustrated in the following quotes:

*‘Percentage of how many women it has worked for.’*

*(Chose MUS)*

*‘It was 90% it worked.’*

*(Chose MUS)*

*‘Better percentage for success’*

*(Chose colposuspension)*

However, 5 of the 53 women stated success rate was *not* the most important factor in their decision-making and, whilst a consideration, avoiding mesh or having the least invasive procedure was their primary concern.

The 53 women who cited efficacy as important to their decision were most likely to opt for a mid-urethral sling (42%) followed by urethral bulking (36%) and colposuspension (23%).

### Duration of recovery

Forty-four women (21%) cited recovery as an important consideration for them when deciding upon a procedure. For many (*n* = 11) this was due to concerns about needing to take time off work. For others, it was due to their responsibilities as a carer within their family (*n* = 6).

*‘I have two toddlers to look after so having an operation was not an option for me at this time. I am a working mum running my own business. My husband works full time. The treatment I have picked is best as I would only be off work for a day.’*

*(Chose urethral bulking)*

Of the women who expressed recovery as a factor which influenced their choice, the majority opted for urethral bulking (*n* = 31, 67%). However, ten chose a mid-urethral sling (22%), two a colposuspension (4%) and one a fascial sling (2%).

### Risk of complications

The risk of post-operative complications was a factor in the decision-making process for 26 women (12%). Most did not specify the post-operative risks they had considered; instead they used generic terms such as: ‘complications’, ‘side-effects’, ‘risks’ and ‘safety’. However, three women from two different sites had concerns regarding post-operative voiding dysfunction and the possibility of self-catheterisation. One woman expressed concerns about pain.

### Risk analysis

Forty women (19%) cited risk as a factor which influenced their decision-making regarding SUI procedures. However, some expressed desire for the procedure they perceived had the lowest risk whilst others described a process of balancing risk with efficacy.

Sixteen women within this group (40%) opted for the procedure they perceived to have the lowest risk as demonstrated in the following quotations:

*‘The safest way.’*

*(Chose urethral bulking)*

*‘I was going to choose the TVT but I work in a solicitors and we have had a lot of cases of women having problems with TVT. This made me change my mind and choose the Bulkamid even though the success rate is not as good. I didn’t want to take the risk at having problems.’*

*(Chose urethral bulking)*

However, a separate subgroup of 18 women (45%) made statements which suggested they had understood and weighed up the risks associated with a procedure but balanced this against other factors in their decision-making:

*‘Low risk with reasonable outcome.’*

*(Chose urethral bulking)*

*‘Success rate, recovery time and after operation complications (this changed my opinion).’*

*(Chose MUS)*

*‘What has the best results and no/minimal side effects.’*

*(Chose colposuspension)*

A further six women (15%) stated they had considered risk; however, they did not clarify whether this influenced them to opt for their perceived lowest risk procedure or whether the risk could be outweighed by other factors such as efficacy.

### Use of mesh

Twenty-four women (11%) stated the use of mesh impacted upon their decision-making. For all but two of these women (*n* = 22, 92%), avoidance of mesh influenced the procedure they opted for. Two women (9%) specified they did not want a foreign body inside them and one woman (4%) had concerns about rejection of mesh.

*‘Colposuspension is using natural own body tissues which hopefully will be less likely to be rejected and there are no tapes so less likely tearing or infected.’*

*(Chose colposuspension)*

Despite acknowledging the risks of mesh procedures, two women (8%) decided to proceed with a mid-urethral sling. Both weighed up the risk of mesh compared with the anticipated effectiveness of the procedure. One woman conducted her own research to enable her to make her decision:

*‘Efficacy/outcome balanced with acceptable risks. Lots of media scare stories about mesh/tape but on reading NICE guidance and the few research documents I could Google, I felt on balance it was a better option long term.’*

*(Chose MUS)*

Another woman had prior history of a mesh procedure and based upon this positive experience wanted mesh again:

*‘Works the most percentage, already had the mesh used in a bowel prolapse, its worked 100% for me.’*

*(Chose MUS)*

### Influence of the clinician

The clinician was cited as a factor which affected decision-making for 20 women (9%). Most of these (*n* = 15) felt they had been advised or guided by the clinician to choose a particular procedure as demonstrated by the following quotes:

*‘The one which the consultant thought would be best for me.’*

*(Chose urethral bulking)*

*‘Done right, trusting the doctors decisions.’*

*(Chose urethral bulking)*

There was evidence some clinicians modified counselling regarding SUI procedures based upon patients’ co-morbidities. Women recognized they had been guided towards a particular procedure by their clinician:

*‘I wasn’t offered any other treatment other than Bulkamid, as I was told I was too overweight for other options.’*

*(Chose urethral bulking BMI-40)*

*‘Advice from doctor (I), listened to the options as I have other medical conditions that affect my treatment options.’*

*(Chose urethral bulking)*

Another four women decided upon their SUI procedure based upon the reputation or expertise of the clinician or hospital they were receiving care from:

*‘The hospital’s reputation and the surgeon, I trusted both on my first meeting.’*

*(Chose urethral bulking)*

*‘(The) Reputation and experience of surgeon in this area carrying out this type of procedure.’*

*(Chose MUS)*

The procedures chosen by women who reported a clinician had influenced their choice were in similar proportions to the overall study population. However, upon comparison of participants depending on their hospital type, those who cited clinician influence at a secondary-care hospital (*n* = 11) were most likely to choose urethral bulking (73% *n* = 8) over a mid-urethral sling (27% *n* = 3). No patients seen at a secondary-care hospital who reported their decision-making was influenced by a clinician chose a colposuspension or fascial sling. At tertiary hospitals, patients who cited that clinicians influenced their choice (*n* = 9) were more evenly spread across all four procedures: urethral bulking (33% *n* = 3), mid-urethral sling (33% *n* = 3), colposuspension (22% *n* = 2) and fascial sling (11% *n* = 1).

### The media

Thirteen women (6%) stated stories in the media had influenced their decision-making:

*‘I was concerned about the safety of the tape being used due to reading about problems in Scotland and watching TV programme highlighting concerns. I opted for colposuspension but after discussing complications…I was persuaded by the consultant to go for the TVT.’*

*(Chose MUS)*

*‘I didn’t want the mesh (TVT) due to press.’*

*(Chose urethral bulking)*

*‘Had opportunity to have the TVT but declined due to negative publicity.’*

*(Chose urethral bulking)*

### Hierarchy of treatments

A novel theme identified amongst women who opted for urethral bulking was the concept of a hierarchical approach to SUI treatments, starting with the perceived least invasive procedure and having alternative procedures if bulking was ineffective. Twelve women (6%) recorded this influenced their decision-making as illustrated in the quotes below:

*‘That I started at the lower end of operations. This would give me future options if needed.’*

*(Chose urethral bulking)*

*‘I prefer to try least invasive method before I would even consider any other. I was told other alternative would not be affected by having the least invasive treatment.’*

*(Chose urethral bulking)*

### Type of anaesthetic

The type of anaesthetic required factored in the decision-making of nine women (4%). All preferred a treatment option which did not require a general anaesthetic:

*‘Don’t like being put to sleep as I am scared.’*

*(Chose urethral bulking)*

*‘I wasn’t very keen on anaesthetic.’*

*(Chose urethral bulking)*

### Other themes

In addition to the themes previously discussed, a small number of women cited different factors which influenced their choice. These included knowing someone who had previously had the procedure (*n* = 6), perceiving the procedure to be definitive treatment (*n* = 5), their plans for further pregnancies (*n* = 5), pain (*n* = 5) and advice from family or friends (*n* = 2).

Despite the questionnaire asking women “What was the most important issue to you when making your treatment decision?” 46 (22%) recorded comments which did not relate to their decision-making or choice of procedure, mentioning only the impact of the condition.

## Discussion

### Main findings

This study was conducted before the pause on the use of tapes was introduced in England. It found when women, in a real clinical situation, across 12 hospitals were offered a choice of four treatments for primary SUI, urethral bulking was the most popular choice (64%). This was despite there being fewer published data on the outcome of bulking compared with the other procedures. There was no association between operation chosen and patient factors such as age, BMI or co-morbidities. There was however a suggestion of a narrow field of choice of procedure in secondary care.

This is the first study to investigate, in real life, the factors women consider important when choosing an operation for primary SUI. The study found that although success was important this was balanced against the perceived “invasiveness” and “risk” of the procedure.

### Interpretation

A recent qualitative study by Casteleijn et al. in which women were asked to choose between two operations, either urethral bulking or a MUS, reported patients would consider urethral bulking as their primary option if it has a cure rate of 70% after 1 year [7]. (However, our study found several women expressed a hierarchical framework in their approach to treatment for SUI. These women first wished to try urethral bulking, which they perceived to be a low-risk simple procedure with short recovery. They accepted treatment may fail and they may need further treatment at a later date. Such hierarchical approaches to treatment are common in other areas of medicine. Before a modern hierarchical treatment approach is promoted for treatment of SUI there is a need for further research to determine not only the overall economic cost and the risk of treatment fatigue but importantly the impact on efficacy of further treatments if the first treatment in the hierarchy fails.

Our study shows the large range of factors influencing women’s decision-making and the highly individual nature of choice. Women balanced the severity of SUI against competing risks of complications and differing success rates, all of which were couched in poorly defined terms such as “invasiveness” and “success”. Understanding the importance of these factors and their meaning to each individual enables clinicians to provide information to women which meets their needs.

Concerns about polypropylene mesh and the media’s representation of mesh procedures were found to have influenced women’s decision-making. It is important to provide women with accurate evidence-based information about mesh complications and complications of other treatment options to ensure they are not required to seek it from unverified sources.

This study has highlighted the complexity of information about SUI procedures being presented to women. Different methods of presenting information, in written and audio visual formats should be explored to ensure we meet patient’s information needs. Factors identified as important to women within this study should be used to develop decision-making tools. The meaning of terms such as risk, success, natural, minor, simple and invasiveness should be explored, qualified and ultimately standardized. The recently published NICE PDA tool [2] demonstrates how little objective evidence is available for each of these complex competing factors. Furthermore, there is no evidence to help women decide on a treatment based on their physical characteristics such as age or BMI. Most women considered a number of factors and tried to weigh these against each other before making their decision. An electronic decision-making tool could help woman to weigh various factors and facilitate their decision-making.

### Limitations

One limitation of the study is that the number of women opting for each procedure was not equal; therefore, there may be a bias in the views expressed. However, the study was designed to ensure it captured a real-life cohort of women undergoing a primary stress incontinence procedure to determine the frequency of their choice.

This research followed a pragmatic design exploring patient choice at a number of different sites; therefore, verbal counselling and written information provided was not standardized. There is therefore the potential that participants were unduly biased towards certain treatments depending on the conscious or unconscious bias of clinicians. Nonetheless, this reflects the real-world nature of the possibility of clinician influence during the process of shared decision-making.

There was a compromise in the study design between capturing information on a large number of women to determine the frequency of choice and the level of detail in the reasons behind their choice. The research team used an open free-text question and thematic analysis technique. This question may not have been sufficiently clear as 25% of responses related to a desire for SUI treatment rather than specifying why they had chosen one procedure over another. Alternatively, this may accurately reflect that for some women the type of treatment is less of a concern than the pressing need for amelioration of their urinary symptoms.

Study eligibility required that all participants be suitable for and offered at least two SUI procedures. Despite this, some participants reported they had only been offered the procedure chosen. Upon retrospective review of these patient’s medical records, it was identified that the clinician had documented discussion of a number of procedures. This demonstrates that a patient’s interpretation of choice may be different from clinicians.

While the study was funded by Contura, who produce Bulkamid, the research was designed and coordinated by the research team with no involvement from the company. There was no financial incentive for clinicians to use Bulkamid nor was Bulkamid provided at a reduced rate for participating centres. Therefore, the authors do not believe the research funding limits the validity of these findings.

## Conclusion

When women were offered a choice of four treatments for primary stress urinary incontinence the most common choice was urethral bulking (64%). A few women appear to value a hierarchical approach to treatment for SUI. There was also evidence the degree of choice varied in different clinical settings, with more variety in procedures chosen at tertiary centres. This may suggest an inherent bias in the counselling that clinicians at secondary-care hospitals offer to their patients. There is also a need to further evaluate the health economic impact and effect on further treatment if a hierarchical approach to treatment is used. Findings from the Latitude study will inform this discussion further.
